# The Role of Omalizumab as an Adjunct to Oral Immunotherapy in Pediatric Food Allergy: A Systematic Review

**DOI:** 10.7759/cureus.85550

**Published:** 2025-06-08

**Authors:** Ahmed Ali Khidir Mustafa, Ehab A Elagab, Ahmed Mohamed AbdElsatar Elhagaly, Afag Abdalla Mohamad Ahmad Bilal, Dilal Haroun Mohamad Hassan, Ayah Adel Ahmed Kamel, Sally Hassan Ali Hassanin

**Affiliations:** 1 Pediatrics, Dr. Mohammad Alfagih Hospital, Riyadh, SAU; 2 Pathology, Najran University, Najran, SAU; 3 Pediatrics, King Fahad Medical City, Riyadh, SAU; 4 Pediatrics, Bedford Hospital, Bedford, GBR; 5 Pediatric Emergency, Dr. Sulaiman Al Habib Medical Group, Jeddah, SAU; 6 Neonatology, St. Peter’s Hospital, Chertsey, GBR; 7 Pediatrics, Alansari Specialized Hospital, Yanbu, SAU

**Keywords:** allergen immunotherapy, anti-ige therapy, food desensitization, ige-mediated allergy, omalizumab, oral immunotherapy, pediatric food allergy

## Abstract

Food allergy represents a growing public health concern, particularly among pediatric populations, where it significantly impacts quality of life and carries the risk of severe reactions. Traditional management through allergen avoidance and emergency medications fails to address the underlying immune dysfunction, prompting interest in active treatments like oral immunotherapy (OIT). However, OIT alone is often limited by safety concerns and variable efficacy, leading to the exploration of adjunctive therapies such as omalizumab treatment to enhance outcomes. This systematic review evaluates the role of omalizumab combined with OIT in pediatric food allergy, aiming to synthesize current evidence on its clinical benefits, safety profile, and potential to induce lasting tolerance. A comprehensive search was conducted across four major databases (PubMed, Scopus, Web of Science, and Embase) following PRISMA guidelines. Studies were included if they investigated omalizumab as an adjunct to OIT in children with immunoglobulin E (IgE)-mediated food allergy and reported measurable clinical outcomes. Two independent reviewers screened records, extracted data, and assessed risk of bias using standardized tools. A narrative synthesis was performed due to heterogeneity in study designs and outcome reporting. The results indicate that omalizumab enhances the safety and efficacy of OIT, with consistent improvements in desensitization rates and reductions in adverse events during treatment. The adjunctive use of omalizumab appears particularly beneficial for high-risk patients and those undergoing multi-allergen OIT, facilitating faster dose escalation and better tolerability. However, evidence on sustained unresponsiveness remains inconsistent, and long-term outcomes require further investigation. The safety profile is favorable overall, with most adverse events being mild to moderate in severity. Omalizumab represents a promising adjunct to OIT in pediatric food allergy, offering a more manageable and effective treatment approach for selected patients. While current evidence supports its use in improving short-term outcomes, additional research is needed to establish standardized protocols, evaluate cost-effectiveness, and clarify its role in achieving long-term tolerance. Clinicians should consider this combined therapy as part of a personalized treatment strategy while remaining mindful of uncertainties and individual patient needs.

## Introduction and background

Food allergy has emerged as a significant and growing public health concern, particularly among pediatric populations, where its prevalence has surged over the past two decades [1]. Affecting nearly 2% of children globally, with notably higher prevalence in Western countries, where rates can exceed 6-8%, food allergies impose a heavy burden on quality of life, psychosocial well-being, and healthcare systems, with potentially life-threatening consequences such as anaphylaxis [2]. Traditional management strategies, including strict allergen avoidance and emergency epinephrine use, remain cornerstones of care, yet these approaches are inherently reactive and fail to address the underlying immune dysregulation [3]. The limitations of such strategies, coupled with the unpredictable nature of accidental exposures, have fueled a paradigm shift toward active treatments that aim to induce desensitization or sustained tolerance. Among these, oral immunotherapy (OIT) has garnered considerable attention for its potential to raise reaction thresholds through incremental allergen ingestion [4]. However, its clinical application is often hindered by safety concerns, including frequent adverse reactions, lengthy protocols, and variable long-term efficacy, leaving clinicians and families grappling with risk-benefit uncertainties [5].

This therapeutic gap has spurred interest in combinatorial approaches that enhance the safety and effectiveness of OIT [6]. Omalizumab, a recombinant humanized monoclonal antibody targeting immunoglobulin E (IgE), binds to circulating IgE and prevents its attachment to high-affinity IgE receptors (FcεRI) on mast cells and basophils, thereby attenuating the allergic inflammatory cascade. By reducing receptor expression and free IgE levels, omalizumab dampens immediate hypersensitivity reactions and stabilizes effector cells involved in allergic responses [3]. Monoclonal antibodies, such as omalizumab, represent a promising adjunct due to their ability to modulate the allergic response by neutralizing free IgE and downregulating high-affinity IgE receptor expression on mast cells and basophils [7]. While not yet universally endorsed in major food allergy guidelines, omalizumab’s off-label use in combination with OIT is gaining traction in clinical research and practice, reflecting growing interest in its therapeutic potential. Omalizumab has demonstrated robust efficacy and effectiveness in a range of allergic conditions, including moderate-to-severe persistent asthma and chronic spontaneous urticaria, where it has been associated with significant reductions in symptom severity, medication use, and exacerbation frequency. These established indications provide a strong foundation for exploring its role in food allergy management [5]. Preclinical and early clinical studies suggest that omalizumab may reduce the frequency and severity of allergic reactions during OIT, potentially accelerating dose escalation and improving adherence [8]. Yet despite a burgeoning body of research, the evidence remains fragmented, with heterogeneous study designs, conflicting outcomes, and unanswered questions about optimal dosing, patient selection, and long-term durability of benefits [9]. For example, studies vary in their use of single versus multiple allergens, omalizumab dosing regimens, and definitions of desensitization success, making direct comparisons challenging. While some trials report dramatic improvements in desensitization rates and safety profiles, others highlight residual risks or limited incremental gains, underscoring the need for a rigorous synthesis of existing data.

This systematic review arrives at a critical juncture, as the integration of biologic therapies into allergy management continues to evolve. By comprehensively analyzing the efficacy, safety, and mechanistic synergies of omalizumab combined with OIT in children, this work seeks to clarify whether this dual approach represents a transformative advancement or a nuanced incremental step. The findings hold immediate relevance for clinicians navigating a rapidly changing therapeutic landscape, policymakers weighing cost-benefit analyses of expensive biologics, and researchers identifying gaps for future investigation. Moreover, they address a fundamental question in pediatric allergy care: Can the strategic pairing of immunomodulators and allergen exposure protocols finally deliver a safer, more reliable path to tolerance - or even cure - for children burdened by the daily realities of food allergy? The answers could redefine standards of care and offer hope to millions of families worldwide.

## Review

Methodology

This systematic review was conducted from 13th March 2025 to 20th April 2025, following the Preferred Reporting Items for Systematic Reviews and Meta-Analyses (PRISMA) 2020 guidelines [10] to evaluate the efficacy and safety of omalizumab as an adjunct to OIT in pediatric food allergy. The methodology was structured to ensure comprehensive identification, rigorous evaluation, and systematic synthesis of all relevant evidence while minimizing bias.

Eligibility Criteria

The eligibility criteria for study inclusion were defined using the PICOS (Population, Intervention, Comparison, Results, and Study Design) framework to ensure a structured and comprehensive selection process. Table 1 outlines both the inclusion and exclusion criteria applied in this review.

**Table 1:** Inclusion and Exclusion Criteria

PICOS Element	Inclusion Criteria	Exclusion Criteria
Population (P)	Pediatric populations (≤18 years) with IgE-mediated food allergy	Studies involving adults or non-IgE-mediated food allergy
Intervention (I)	Omalizumab used as an adjunct to oral immunotherapy (OIT)	Studies not involving omalizumab or OIT
Comparison (C)	Not mandatory; studies with or without a comparison group were eligible	-
Outcomes (O)	Studies reporting measurable outcomes such as desensitization rates; sustained unresponsiveness; adverse events	Studies not reporting relevant clinical outcomes
Study Design (S)	Randomized controlled trials (RCTs); non-randomized controlled trials; prospective observational studies	Case reports; narrative reviews; conference abstracts without peer-reviewed full-text publications
Other Criteria	No restrictions on publication date or language	-

Information Sources and Search Strategy

A systematic search was conducted across four major databases - PubMed, Scopus, Web of Science, and Embase - from their inception to the latest available date. The search strategy incorporated controlled vocabulary (Medical Subject Headings (MeSH), Emtree) and free-text terms related to anti-IgE therapy, OIT, and food allergy. No date restrictions were used while searching for relevant studies. The PubMed search strategy, outlined in Table 2, was adapted for syntax variations in other databases.

**Table 2:** PubMed Search Strategy OIT, Oral Immunotherapy

Concept	Search Terms
Anti-IgE Therapy	"Omalizumab" OR "anti-IgE" OR "Xolair" OR "IgE inhibitor"
Oral Immunotherapy	"Oral immunotherapy" OR "OIT" OR "food desensitization"
Food Allergy	"Food allergy" OR "peanut allergy" OR "milk allergy" OR "egg allergy"
Pediatric Population	"Child" OR "pediatric" OR "adolescent" OR "infant"
Combined Search	#1 AND #2 AND #3 AND #4

Similar search strategies were employed in Scopus, Web of Science, and Embase, with adjustments for database-specific terminologies and syntax rules.

Study Selection Process

The study selection adhered to the PRISMA framework and involved two sequential screening phases. First, two independent reviewers screened titles and abstracts to exclude irrelevant records. Second, full-text articles of potentially eligible studies were assessed against the predefined inclusion criteria. Disagreements between reviewers were resolved through discussion or consultation with a third reviewer to ensure consensus. The screening process was documented in a PRISMA flow diagram, detailing the number of records identified, excluded, and included at each stage.

Data Extraction and Management

A standardized data extraction form was developed to systematically capture key study characteristics and outcomes. Two reviewers independently extracted data on study design, participant demographics (e.g., age, allergen type, baseline IgE levels), intervention protocols (e.g., omalizumab dosing, OIT regimen, treatment duration), and primary outcomes (e.g., desensitization success, adverse events (AEs)). Extracted data were cross-verified for accuracy, and discrepancies were resolved through iterative discussion.

Risk of Bias Assessment

The methodological quality of the included randomized controlled trials was assessed using the Cochrane Risk of Bias 2 (RoB 2) tool [11]. This tool evaluates potential bias across five specific domains: (1) bias arising from the randomization process, (2) bias due to deviations from intended interventions, (3) bias due to missing outcome data, (4) bias in measurement of the outcome, and (5) bias in selection of the reported result. Each domain was judged as having a “low risk,” “some concerns,” or “high risk” of bias based on signaling questions and standardized criteria. An overall risk of bias judgment was then assigned to each study. Two reviewers independently conducted the assessments, and any disagreements were resolved through discussion or consultation with a third reviewer.

Data Synthesis and Analysis

Given the clinical and methodological heterogeneity among studies (e.g., variations in allergens, OIT protocols, and outcome definitions), a narrative synthesis was performed. Data were organized by key themes, such as desensitization efficacy, safety profiles, and sustained unresponsiveness (SU). We did not conduct a meta-analysis due to significant clinical and methodological heterogeneity among the included studies, including variations in study designs, allergen types, OIT protocols, and outcome measures. The lack of standardized reporting for key outcomes such as desensitization criteria and AE definitions further limited the feasibility of quantitative synthesis. Therefore, a narrative synthesis was deemed more appropriate to comprehensively interpret the findings while acknowledging the diversity in the evidence base. To enhance the transparency of the synthesis process, studies were coded and grouped thematically according to outcome categories. A comparative matrix was then developed to identify patterns and variations across studies within each theme.

Ethical Considerations

As this review synthesized existing published data, no additional ethical approval was required. However, all included studies were reviewed to confirm adherence to ethical standards in their original research, including informed consent and institutional review board approvals where applicable.

Results

Study Selection Process

**Figure 1:**
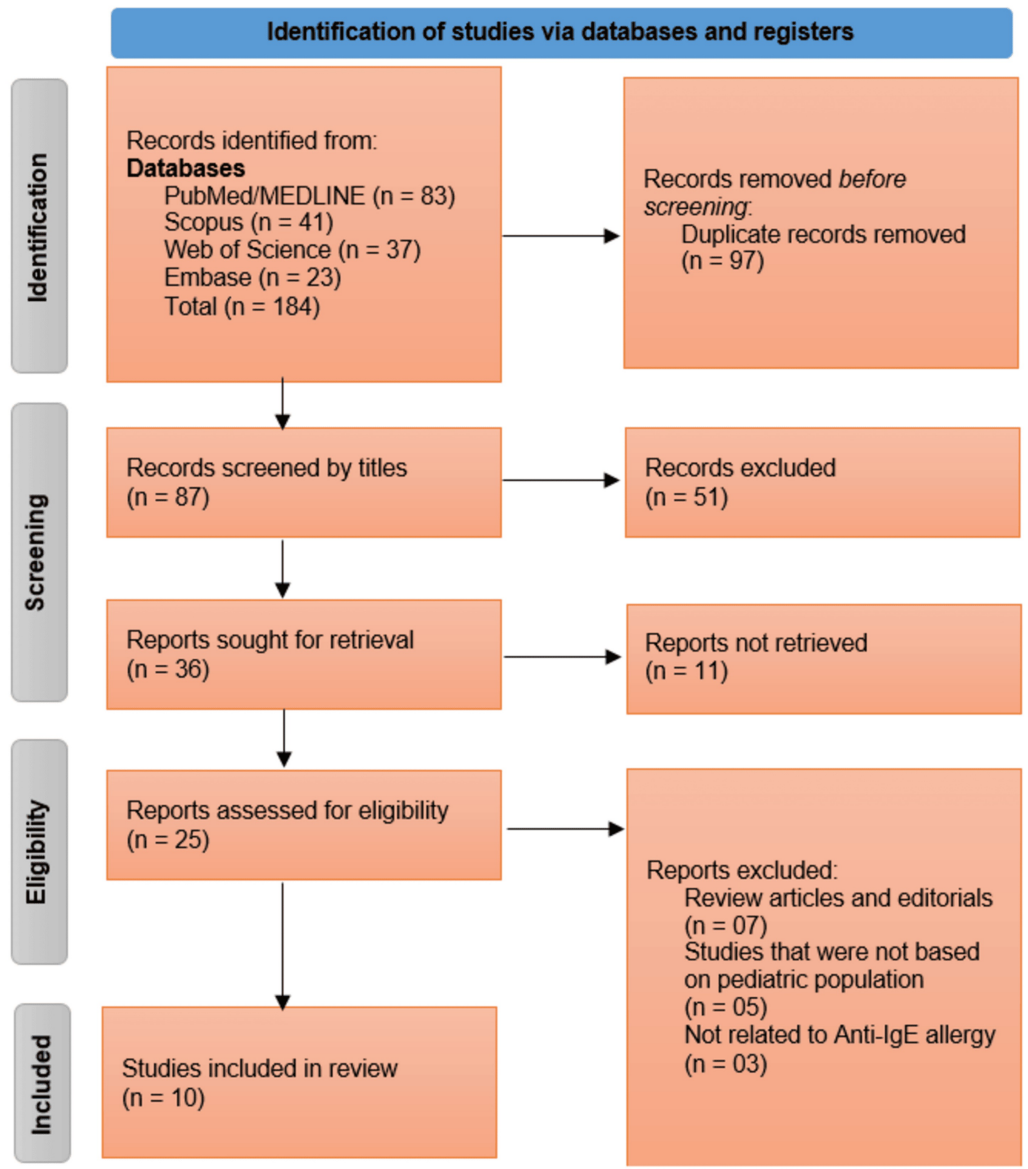
PRISMA Flowchart Illustrating the Studies Selection Process for This Systematic Review PRISMA, Preferred Reporting Items for Systematic Reviews and Meta-Analyses

The systematic search across four databases - PubMed/MEDLINE (n = 83), Scopus (n = 41), Web of Science (n = 37), and Embase (n = 23) - yielded 184 records, of which 97 duplicates were removed prior to screening. The remaining 87 records underwent title and abstract screening, resulting in the exclusion of 51 irrelevant studies. Of the 36 reports sought for full-text retrieval, 11 were unavailable, leaving 25 studies for eligibility assessment. After excluding review articles (n = 7), studies not focused on pediatric populations (n = 5), and those unrelated to omalizumab (n = 3), a total of 10 studies met the inclusion criteria and were incorporated into the systematic review (Figure 1).

Study Characteristics and Design

The systematic review included 10 studies [12-21] investigating the role of omalizumab as an adjunct to OIT in pediatric food allergy, with varying designs, sample sizes, and allergen targets (Table 3). The studies spanned multiple countries, including the USA, Canada, Japan, Italy, and Sweden, and employed diverse methodologies, ranging from RCTs to open-label pilot studies. Sample sizes varied significantly, from small cohorts of four participants [18] to larger trials like Langlois et al. [12], which enrolled 72 children. The most commonly studied allergens were peanut [19,20], cow’s milk [16,17], and multi-food allergies [13,15]. Omalizumab dosing regimens also differed, with durations ranging from eight weeks [13] to 24 weeks [17], and some studies employed individualized tapering [20].

**Table 3:** Summary and Key Findings of Studies AE, Adverse Event; ABA, Allergen-Binding Activity; Anti-IgE, Anti-Immunoglobulin E; CD-sens, Basophil Allergen Threshold Sensitivity Test; DBPCFC, Double-Blind Placebo-Controlled Food Challenge; IgE, Immunoglobulin E; IgG4, Immunoglobulin G4; NR, Not Reported; OFC, Oral Food Challenge; OIT, Oral Immunotherapy; QoL, Quality of Life; RCT, Randomized Controlled Trial; SU, Sustained Unresponsiveness; sIgE, Specific Immunoglobulin E

Study (Author, Year)	Country	Study Design	Sample Size (Anti-IgE + OIT/Control)	Food Allergen(s)	Type of Anti-IgE Used	Duration of Therapy	Outcome Measures	Key Findings
Langlois et al., [12] 2020	Canada	Randomized, placebo-controlled trial	72 (36 on 8 mg/kg Omalizumab + OIT, 36 on 16 mg/kg Omalizumab + OIT)/18 (Placebo + OIT)	Multiple (3 or more)	Omalizumab	20 weeks (12 weeks full dose, 4 weeks at 50%, 4 weeks at 25%)	Time from initial food escalation to 1500 mg protein maintenance dose	The study aims to determine the dose-related effect of omalizumab on accelerating OIT. Uses a symptom-driven, patient-centered up-dosing algorithm to assess treatment effectiveness speed and safety.
Bégin et al., [13] 2014	USA	Phase 1 clinical trial (single site)	25/0	Multiple (up to 5 allergens simultaneously)	Omalizumab	8 weeks before + 8 weeks after OIT (16 weeks total)	Safety, dose tolerability, home reaction rate, achievement of maintenance dose	94% reactions were mild; 1 severe reaction; 76% tolerated initial escalation; median time to maintenance dose 18 weeks at 4,000 mg per allergen.
Wood et al., [14] 2022	United States	Phase 3 randomized clinical trial	Not specified (still enrolling)	Peanut + at least 2 of milk, egg, wheat, cashew, hazelnut, walnut	Omalizumab	NR	Safety and efficacy of omalizumab alone vs. omalizumab + OIT; long-term desensitization outcomes	Study ongoing; aims to assess omalizumab as monotherapy and as adjunct to multiallergen OIT
Andorf et al., [15] 2018	USA (Stanford)	Blinded phase 2 clinical trial	36 (anti-IgE + OIT), 12 (placebo), 12 (untreated control)	Multiple foods (2-5 per participant)	Omalizumab	16 weeks of omalizumab; OIT started at week 8 and both stopped by week 16; follow-up until week 36	Proportion passing DBPCFC to ≥2 foods; % OIT doses with adverse events	83% in omalizumab group passed DBPCFC vs. 33% in placebo (P = 0.0044); significantly fewer adverse events in omalizumab group; no serious AEs reported
Wood et al., [16] 2016	USA	Randomized, double-blind, placebo-controlled trial	27/28	Milk	Omalizumab	4 months pre-OIT + 22-40 weeks escalation + maintenance until month 28; SU tested at month 32	Desensitization at month 28; Sustained Unresponsiveness (SU) at month 32; Adverse reactions during OIT	Omalizumab significantly reduced adverse reactions during OIT escalation; no statistically significant improvement in desensitization or SU rates
Takahashi et al., [17] 2017	Japan	Prospective randomized controlled trial	10 (OIT + omalizumab)/6 (control)	Cow’s milk	Omalizumab	24 weeks	Primary: Desensitization at 8 weeks post-OMB discontinuation; Skin prick test reactivity	100% of OIT + OMB group desensitized vs. 0% in control (P < 0.001); significant reduction in skin prick test wheal diameter in OIT + OMB group (P < 0.05)
Badina et al., [18] 2022	Italy	Prospective observational	4/0	Cow's milk	Omalizumab	8 weeks pre-OFC + 12 months during OIT	OFC threshold, sIgE, IgG4, QoL	All patients increased CM threshold, tolerated OIT with minimal/mild symptoms, improved QoL, ↑ milk-specific IgG4
MacGinnitie et al., [19] 2017	USA	Randomized controlled trial	29 (omalizumab + OIT)/8 (placebo + OIT)	Peanut	Omalizumab	12 weeks pre-OIT + up to 6 weeks during OIT (18 weeks total)	Peanut dose tolerated during initial desensitization. Success rate of 2000 mg maintenance - 4000 mg open food challenge- reaction rates	Omalizumab allowed rapid desensitization to 2000 mg peanut in 79% vs. 12% in placebo. 4000 mg challenge passed by 23/29 vs. 1/8. Reaction rates not significantly lower, but exposure was higher in treated group
Brandström et al., [20] 2019	Sweden	One-armed open phase-2 trial	23/0	Peanut	Omalizumab	Individualized duration; OIT up-dosed over 8 weeks, followed by 12 weeks maintenance and omalizumab tapering	CD-sens test, allergen-binding activity (ABA), specific IgE/IgG/IgG4 to peanut and components, clinical symptoms, open peanut challenge	All 23 reached 2800 mg dose. 48% continued OIT after stopping omalizumab. Fewer adverse events during full-dose omalizumab. Greater IgG4 increase and lower baseline IgE and CD-sens predicted success.
Schneider et al., [21] 2013	USA	Open-label single-arm interventional pilot	13 (13 anti-IgE + OIT; no control group)	Peanut	Omalizumab (Xolair)	Omalizumab administered as a pretreatment and during the desensitization phase (median 8 weeks to reach maintenance dose), followed by continuation of OIT	Tolerance of escalating peanut doses (initial desensitization, maintenance dose achievement, challenge tolerance); safety (reaction severity grading)	Rapid desensitization achieved; most reached 4000 mg dose; reactions were mostly mild and manageable

Efficacy of Anti-IgE Therapy in Accelerating and Enhancing OIT

The adjunct use of omalizumab demonstrated promising efficacy in accelerating desensitization and improving OIT outcomes. In the RCT by Andorf et al. [15], 83% of participants receiving omalizumab + OIT passed a double-blind, placebo-controlled food challenge (DBPCFC) to ≥2 foods, compared to only 33% in the placebo group (p = 0.0044). Similarly, Takahashi et al. [17] reported a 100% desensitization rate in the omalizumab-OIT group versus 0% in controls (P < 0.001), with significantly reduced skin prick test reactivity. For peanut allergy, MacGinnitie et al. [19] found that 79% of omalizumab-treated participants achieved rapid desensitization to 2000 mg peanut protein, compared to 12% in the placebo group, with 23/29 later tolerating 4000 mg. Notably, Brandström et al. [20] achieved a 100% desensitization rate to 2800 mg peanut in their open-label trial, with nearly half maintaining tolerance post-omalizumab withdrawal. 

Time to desensitization was also reduced in several studies. Bégin et al. [13] reported a median time of 18 weeks to reach maintenance dosing in a multi-allergen OIT protocol, while Schneider et al. [21] observed a median of eight weeks to achieve a 4000 mg peanut dose. However, not all studies showed statistically significant improvements in SU. Wood et al. [16] noted higher SU rates with omalizumab (48.1% vs. 35.7% placebo), but this difference did not reach significance, suggesting that while anti-IgE therapy may hasten desensitization, its long-term immunomodulatory effects require further investigation. 

Safety and Tolerability Profile

The safety data from included studies were generally favorable, with most AEs being mild to moderate (Table 4). Andorf et al. [15] reported fewer AEs in the omalizumab group (27%) compared to placebo (68%), predominantly gastrointestinal symptoms, with no severe or grade ≥3 reactions. Similarly, Wood et al. [16] observed lower reaction rates during OIT escalation in omalizumab-treated participants (2.1% of doses vs. 16.1% in placebo). Mild symptoms like oral itching and transient abdominal pain were common [18,21], but severe reactions were rare, with only one severe event reported across all studies [13]. 

**Table 4:** Summary of Clinical Outcomes and Adverse Events AE, Adverse Event; IFE, Initial Food Escalation; NR, Not Reported; OIT, Oral Immunotherapy; OMB, Omalizumab; OML, Omalizumab; OR, Odds Ratio; TBD, To Be Determined

Study (Author, Year)	Desensitization Rate (%)	Sustained Unresponsiveness (%)	Time to Desensitization	Rate of Adverse Events (%)	Type of Adverse Events	Withdrawal Rate (%)
Langlois et al., [12] 2020	NR	NR	Primary endpoint: time from IFE to target dose (1500 mg) - TBD	NR	NR - safety monitored, no specific events listed	NR
Bégin et al., [13] 2014	Not explicitly stated; implied high (76% tolerated initial escalation dose)	NR	Median 18 weeks	5.3% per dose administered	94% mild, 1 severe reaction reported; symptoms not detailed	0% (no withdrawals mentioned)
Wood et al., [14] 2022	Not reported (study ongoing)	NR	NR	NR	NR	NR
Andorf et al., [15] 2018	83% (omalizumab group) vs. 33% (placebo)	NR	28 weeks (oral immunotherapy started at week 8, challenge at week 36)	27% (omalizumab group) vs 68% (placebo)	Mostly gastrointestinal; no serious or grade ≥3 AEs	0% (no withdrawals)
Wood et al., [16] 2016	88.9% (omalizumab) vs. 71.4% (placebo)	48.1% (omalizumab) vs. 35.7% (placebo)	22-40 weeks to maintenance (plus 4 months lead-in)	2.1% of doses (omalizumab) vs 16.1% (placebo) caused symptoms	Dose-related reactions; some requiring treatment	NR
Takahashi et al., [17]2017	100% (10/10 in OMB-OIT group)	NR	8 weeks after OMB discontinuation (32 weeks total)	NR	NR	0% (no withdrawals reported)
Badina et al., [18] 2022	Not explicitly reported (all patients increased threshold)	Suggestive (patients maintained tolerance long after OML stopped)	Not clearly specified (OML given for 8 weeks pre-OIT, then 12 months with OIT)	0-25% (1 of 4 patients had mild symptoms)	Oral itching, mild abdominal pain (transient)	0%
MacGinnitie et al., [19] 2017	79%	79% (23/29 passed 4000 mg challenge)	~8 weeks	Not significantly different (OR 0.57; P = 0.15)	Not specified (implied to be reactions during OIT)	NR
Brandström et al., [20] 2019	100% (23/23 reached 2800 mg)	48% (11/23 continued post-omalizumab)	8 weeks to 2800 mg maintenance	Not quantified; noted as rare during full-dose omalizumab, increased after withdrawal	Moderate/systemic allergic reactions (rare during full-dose omalizumab; more frequent after withdrawal)	0% (no reported withdrawals)
Schneider et al., [21] 2013	92% (12/13 reached 4000 mg)	NR	Median of 8 weeks	100% (13/13 experienced some reaction)	6 mild/no reaction, 5 grade 2, 2 grade 3 (all responsive to treatment)	0%

Withdrawal rates due to AEs were notably low, with no dropouts reported in most trials [15,17,20]. However, some studies noted increased reaction frequency after omalizumab discontinuation [20], highlighting the need for careful monitoring during therapy transitions. 

Heterogeneity and Limitations

Despite encouraging results, heterogeneity in study designs, outcome measures, and allergen protocols limits direct comparisons. For instance, Langlois et al. [12] focused on dose-related efficacy but did not report desensitization rates, while Wood et al. [14] is ongoing, with outcomes pending. Additionally, small sample sizes in some studies [17,18] may underpower subgroup analyses. The variability in SU assessment timelines further complicates conclusions about long-term tolerance.

Summary of Risk of Bias Findings

The Cochrane RoB 2 assessment revealed that four RCTs [12,15,16,19] had a low risk of bias, demonstrating robust randomization, adherence to protocols, low attrition, and blinded outcome assessment. Takahashi et al. [17] raised some concerns due to unclear randomization and lack of blinding in the control group. The remaining studies - Bégin et al. [13], Badina et al. [18], Brandström et al. [20], and Schneider et al. [21] - were non-randomized or single-arm trials, resulting in a high risk of bias primarily from open-label designs, lack of comparators, and potential performance bias. The ongoing Wood et al. [16] trial could not be fully assessed. Overall, while the RCTs provide reliable evidence, the inclusion of high-risk studies necessitates cautious interpretation of pooled efficacy and safety outcomes (Table 5).

**Table 5:** Summary of Risk of Bias

Study (Author, Year)	Randomization Process	Deviations From Intended Interventions	Missing Outcome Data	Measurement of Outcomes	Selection of Reported Results	Overall Risk of Bias
Langlois et al., [12] 2020	Low	Low	Low	Low	Low	Low
Bégin et al., [13] 2014	N/A (Single-arm)	N/A	Low	High (Open-label)	Low	High
Wood et al., [14] 2022	Unclear (Ongoing)	Unclear	Unclear	Unclear	Unclear	Unclear
Andorf et al., [15] 2018	Low	Low	Low	Low	Low	Low
Wood et al., [16] 2016	Low	Low	Low	Low	Low	Low
Takahashi et al., [17] 2017	Some concerns	Low	Low	High (Unblinded control)	Low	Some concerns
Badina et al., [18] 2022	N/A (Observational)	N/A	Low	High (Open-label)	Low	High
MacGinnitie et al., [19] 2017	Low	Low	Low	Low	Low	Low
Brandström et al., [20] 2019	N/A (Single-arm)	N/A	Low	High (Open-label)	Low	High
Schneider et al., [21] 2013	N/A (Single-arm)	N/A	Low	High (Open-label)	Low	High

Discussion

This study provides compelling evidence regarding the potential benefits of combining omalizumab with OIT for pediatric food allergy, while also highlighting critical gaps and inconsistencies in the current evidence base. Across the 10 included studies, omalizumab demonstrated a consistent ability to enhance the safety and efficacy of OIT, particularly in accelerating desensitization and reducing adverse reactions during dose escalation. For instance, Andorf et al. [15] reported significantly higher desensitization rates (83% vs. 33%) in participants receiving omalizumab plus OIT compared to placebo, while Takahashi et al. [17] achieved 100% desensitization in their treatment group, underscoring the potential of this combined approach. These results align with earlier mechanistic studies showing that omalizumab reduces mast cell and basophil reactivity by lowering free IgE levels, thereby creating a more permissive environment for allergen exposure [22]. However, the variability in SU outcomes - such as the modest 48.1% SU rate in Wood et al. [16] versus the 79% reported by MacGinnitie et al. [19] - suggests that the long-term immunomodulatory effects of anti-IgE therapy remain incompletely understood. This discrepancy may reflect differences in study design, allergen types, or follow-up durations, emphasizing the need for standardized protocols to evaluate SU in future trials. 

The safety profile of omalizumab-adjuvanted OIT appears favorable overall, with most studies reporting mild to moderate AEs, such as transient gastrointestinal symptoms or oral pruritus [13, 18]. Notably, severe reactions were rare, and withdrawal rates due to AEs were negligible across studies, reinforcing the feasibility of this approach in clinical practice. These findings contrast with standalone OIT trials, where higher rates of systemic reactions (e.g., anaphylaxis) have been documented [23]. The reduction in AEs with omalizumab is particularly significant for multi-allergen OIT, as demonstrated by Bégin et al. [13], where 76% of participants tolerated initial escalation doses despite simultaneous exposure to up to five allergens. This aligns with real-world data from Schneider et al. [21], where rapid desensitization to peanut was achieved with minimal grade 3 reactions. However, the increased frequency of reactions post-omalizumab withdrawal in Brandström et al. [20] raises important questions about the durability of protection and the optimal duration of therapy, which warrant further investigation. 

When compared to existing literature, our review corroborates the growing consensus that omalizumab mitigates the risks of OIT, but its role in inducing lasting tolerance remains uncertain. For example, a meta-analysis by Nurmatov et al. [24] concluded that OIT alone increases the likelihood of desensitization but carries a high burden of AEs, while more recent work by Chu et al. [25] suggested that omalizumab may bridge this safety-efficacy gap. Our findings extend these observations by highlighting the heterogeneity in outcome measures, such as the use of different maintenance doses (e.g., 300 mg vs. 4000 mg protein) and variable definitions of SU, which complicate cross-study comparisons. This inconsistency mirrors challenges noted in broader OIT research, where a lack of harmonization in protocols has been a persistent barrier to evidence synthesis [26].

The mechanistic advantages of omalizumab are further supported by biomarker data from included studies. For instance, Brandström et al. [20] reported increases in allergen-specific IgG4 and reductions in basophil reactivity, consistent with the immunomodulatory effects observed in asthma and chronic urticaria [27]. However, the absence of robust correlations between these biomarkers and clinical outcomes in some studies [16] suggests that additional factors, such as T-regulatory cell function or epithelial barrier integrity, may influence treatment success. This complexity underscores the need for integrated translational research to identify predictive biomarkers, as proposed by Santos et al. [28] in their work on personalized allergy therapeutics. 

Despite these promising results, the review reveals critical limitations in the current evidence base. Most notably, the predominance of small, single-center trials [18,21] and the lack of long-term follow-up data limit the generalizability of findings. Only one ongoing trial [14] aims to evaluate multi-allergen OIT with omalizumab in a large cohort, highlighting the need for more rigorous, multicenter studies. Furthermore, the exclusion of non-English studies and potential publication bias may have skewed the evidence toward positive outcomes, a limitation acknowledged in prior reviews. 

Limitations

The primary limitations of this review include the heterogeneity of study designs, variations in outcome reporting, and the inclusion of non-randomized trials with inherent bias. While RCTs like Andorf et al. [15] and MacGinnitie et al. [19] provided high-quality evidence, the reliance on open-label studies [20] introduces performance bias. Additionally, the lack of standardized protocols for assessing SU and the paucity of data on cost-effectiveness represent gaps that future research must address. 

## Conclusions

Omalizumab combined with OIT represents a significant advancement in pediatric food allergy treatment, offering improved safety and efficacy compared to OIT alone, particularly for high-risk patients and those with multiple food allergies. While the evidence clearly shows enhanced desensitization rates and reduced adverse reactions during treatment, questions remain regarding the long-term durability of tolerance and optimal treatment protocols, as well as the economic implications of this combined approach. The current body of evidence suggests that omalizumab-adjuvanted OIT should be considered as a valuable therapeutic option for carefully selected patients, though further research is needed to standardize outcome measures, establish cost-effectiveness, and investigate potential synergies with emerging therapies. Ultimately, this combined approach moves us closer to more effective food allergy management, but clinicians must weigh the benefits against remaining uncertainties when making treatment decisions, with an emphasis on personalized care and shared decision-making with patients and families. Future studies should focus on clarifying the mechanisms of sustained tolerance and developing predictive biomarkers to optimize patient selection and treatment outcomes.
